# Biomarkers of Functional Decline, Morbidity and Mortality

**DOI:** 10.1093/geroni/igaf122.783

**Published:** 2025-12-31

**Authors:** Keisha Lovence

**Affiliations:** University of Pittsburgh, Pittsburgh, Pennsylvania, United States

## Abstract

Research in this session will describe clinical and biological markers for functional decline, morbidity and mortality.

